# The predictive value of RDW in AKI and mortality in patients with traumatic brain injury

**DOI:** 10.1002/jcla.23373

**Published:** 2020-08-25

**Authors:** Ruo Ran Wang, Min He, Xiao Feng Ou, Xiao Qi Xie, Yan Kang

**Affiliations:** ^1^ Department of Critical Care Medicine West China Hospital Sichuan University Chengdu China

**Keywords:** acute kidney injury, brain trauma, marker, red blood cell distribution width

## Abstract

**Background:**

Red blood cell distribution width (RDW) has been validated valuable in predicting outcome and acute kidney injury (AKI) in several clinical settings. The aim of this study was to explore whether RDW is associated with outcome and AKI in patients with traumatic brain injury (TBI).

**Methods:**

Patients admitted to our hospital for TBI from January 2015 to August 2018 were included in this study. Multivariate logistic regression analysis was performed to identify risk factors of AKI and outcome in patients with TBI. The value of RDW in predicting AKI and outcome was evaluated by receiver operating characteristic (ROC) curve.

**Results:**

Three hundred and eighteen patients were included in this study. The median of RDW was 14.25%. We divided subjects into two groups based on the median and compared difference of variables between two groups. The incidence of AKI and mortality was higher in high RDW (RDW > 14.25) group (31.45% vs 9.43%, *P* < .001; 69.81% vs 29.56%, *P* < .001). Spearman's method showed RDW was moderately associated with 90‐day Glasgow Outcome Scale (GOS) (*P* < .001). In multivariate logistic regression analysis, RDW, lymphocyte, chlorine, and serum creatinine were risk factors of AKI. And Glasgow Coma Scale (GCS), glucose, chlorine, AKI, and RDW were risk factors of mortality. The area under the ROC curve (AUC) of RDW for predicting AKI and mortality was 0.724 (0.662‐0.786) and 0.754 (0.701‐0.807), respectively. Patients with higher RDW were likely to have shorter median survival time (58 vs 70, *P* < .001).

**Conclusions:**

Red blood cell distribution width is an independent risk factor of AKI and mortality in patients with TBI.

## 1 INTRODUCTION

Traumatic brain injury (TBI) has attracted much attention for the severe burden it brings to the public health and social economy. More than 50 million people suffer TBI each year in the world.^1^ And it has become the primary cause of mortality in people under 40 years.^2^ Even if patients suffered from TBI survive, their daily activities would be strictly limited and quality of life would be severely impaired.^3^ It has been demonstrated that not only the primary and secondary brain injury, but also the non‐neurologic organ dysfunction following TBI would contribute to the increased risk of mortality.^4^ Acute kidney injury (AKI), one of non‐neurologic complications following TBI, has attracted much attention because of its effect on the increased mortality.^5^, ^6^, ^7^, ^8^ Therefore, identifying patients who are at high risk of developing AKI and taking suitable treatment strategies may be beneficial for recovery and prognosis of patients.

Red blood cell distribution width (RDW) usually plays an important role in the diagnosis and classification of anemia. However, it has been regarded as an indicator of inflammation, recently. A growing number of studies evaluate the prognostic value of RDW in various clinical settings including critical illness, acute pancreatitis, cancer, diabetes mellitus, and stroke.^9^, ^10^, ^11^, ^12^, ^13^ There are two studies demonstrating the association between RDW and mortality in patients with TBI. However, in consideration of the value of RDW in predicting the prognosis of TBI, these two studies have shown conflicting conclusions.^14^, ^15^ It is unknown whether RDW can really act as a valuable marker in predicting outcome of patients with TBI.

In addition to the prognostic value, RDW also has been confirmed valuable in predicting AKI in many clinical settings. Recent studies report that RDW is associated with AKI in patients with sepsis, after cardiac surgery, and those hospitalized in coronary care unit, respectively.^16^, ^17^, ^18^ However, the predictive value of RDW in AKI following TBI has not been validated. We design this study to confirm whether RDW is helpful in predicting the outcome of patients with TBI and discover the predicting value of RDW in AKI following TBI.

## 2 MATERIALS AND METHODS

### 2.1 Patients

**Figure 1:**
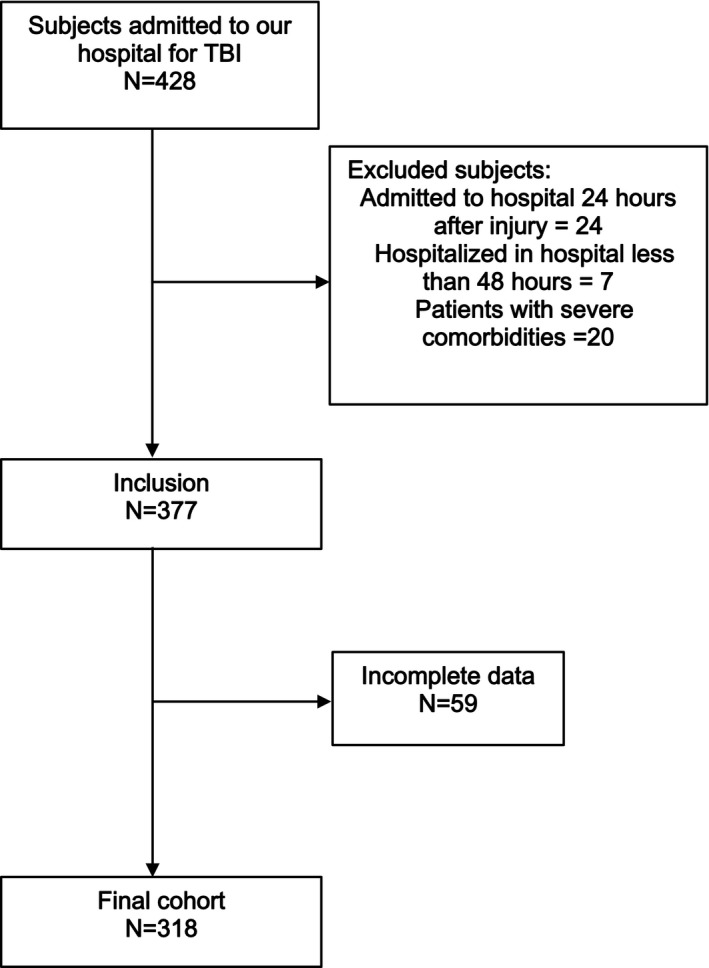
Flowchart of participant recruitment

We conducted this retrospective study in West China Hospital affiliated to Sichuan University. A total of 318 patients admitted for TBI between January 2015 and August 2018 were included in our study. The inclusion criteria of participants were showed as follows: (a) patients diagnosed with TBI and transferred to our hospital within 24 hours after injury; (b) patients hospitalized in the neurosurgery department or neuro‐intensive care unit (NICU) for more than 48 hours. These patients were excluded from this study: (a) patients had a history of cardiovascular diseases, hematological system diseases, metabolic diseases, chronic hepatorenal diseases, and cancer; (b) patients whose clinical records and laboratory tests were not complete and who did not follow treatment strategies (Figure 1). And we considered it was unnecessary to acquire informed consent because of this study design.

### 2.2 Data collection

Blood samples of patients were regularly collected once they admitted to our hospital. All laboratory and clinical data were collected from our electronic medical record (EMR) system. We selected results of laboratory test on admission as included variables. The GCS score on admission was evaluated immediately by experienced emergency physician and neurosurgeons. The diagnosis of TBI was confirmed by imaging tests including CT and MRI. We recorded operations and shock experienced within 48 hours of admission. AKI was diagnosed based on meeting any of the 2012 KIDGO criteria 48 hours after admission: (a) increase in serum creatinine (SCr) by ≥26.5 μmol/L within 48 hours, (b) increase of SCr of ≥1.5 times over baseline (which is known or presumed within the prior 7 days), and (c) urine volume < 0.5 mL/kg/h for 6 hours. The survival outcome and GOS of patients were obtained through medical record or telephone following up until 3 months after initial injury.

### 2.3 Statistical analysis

Kolmogorov‐Smirnov test was used to testify the normality of included variables. Categorical data were showed as numbers (percentage). Normal distribution data were showed as mean ± standard deviation while non‐normal distribution data were showed as median (interquartile range). The difference between two normal distribution variables was testified using independent Student's *t* test. And the difference between non‐normal distribution variables was compared using Mann‐Whitney *U* test. Chi‐square test was used to analyze the difference between categorical variables. We used univariate and multivariate logistic regression analysis to discover potential risk factors of AKI and mortality. The odds ratio (OR) and 95% confidence intervals (CI) of all risk factors were calculated. We also calculated Spearman's correlation coefficients for the relationships between RDW and other clinical or laboratory factors. ROC curves were generated to evaluate predictive value of RDW in AKI and mortality. Meanwhile, the sensitivity, specificity, and cutoff value were calculated. In addition, we divided patients into two groups based on the cutoff value and then draw cumulative survival curves of two groups using Kaplan‐Meier analysis. The difference of survival curves between two groups was compared by log‐rank test.

A *P* value < .05 was considered statistically significant. SPSS 22.0 Windows software (SPSS, Inc) was used for all statistical analyses.

## 3 RESULTS

### 3.1 Baseline characteristics of patients

The median of RDW was 14.25%. We divided patients into two groups including high RDW (RDW > 14.25%) group and low RDW (RDW < 14.25%) group. As we can see in Table 1, the age of high RDW group was higher than low RDW group (47 vs 41, *P* < .001). The mean arterial pressure (MAP) of two groups was not significantly different (89.33 vs 91, *P* = .838). Patients in high RDW group had higher occurrence of shock than low RDW group (61 vs 37, *P* = .003). And GCS of high RDW group was lower than that of low RDW group (5 vs 7, *P* = .001) which indicated that patients with high RDW were more severe. Considering laboratory tests, we found glucose, chlorine, serum urea, and creatinine were higher in high RDW group with statistical significance. Instead, platelet, lymphocyte, hemoglobin, albumin, and phosphate were significantly lower in high RDW group. In addition, patients with high RDW were more likely to undergo decompressive craniectomy and hematoma evacuation (37.74% vs 22.64%, *P* = .003; 42.14% vs 27.67%, *P* = .007). Finally, we found the important fact that the occurrence of AKI and mortality was higher in high RDW group (31.45% vs 9.43%, *P* < .001; 69.81% vs 29.56%, *P* < .001). The 90‐day GOS and length of hospital stay (LOS) were both lower in high RDW group (1 vs 3, *P* < .001; 8 vs 18, *P* < .001).

**Table 1:** Baseline characteristics of patients with RDW > 14.25% and RDW < 14.25%

	Total (n = 318)	CV > 14.25% (n = 159)	CV < 14.25% (n = 159)	*P* value
Age (year)	45 (28‐58)	47 (33‐62)	41 (26‐53)	<.001
Male	245 (77.04%)	116 (72.96%)	129 (81.13%)	.082
Injury cause				
Traffic accident	211 (66.35%)	112 (70.44%)	99 (62.26%)	.123
Failing injury	82 (25.79%)	37 (23.27%)	45 (28.30%)	.305
Others	25 (7.86%)	10 (6.29%)	15 (9.43%)	.296
MAP (mmHg)	90 (77.25‐102)	89.33 (76.33‐102.67)	91 (78.67‐101)	.838
Shock	98 (30.82%)	61 (38.36%)	37 (23.27%)	.003
GCS	6 (5‐8)	5 (5‐7)	7 (5‐11)	.001
Laboratory tests				
WBC (10^9^/L)	14.66 (10.53‐19.23)	14.84 (10.8‐19.45)	14.34 (10.36‐19.14)	.767
Neutrophil (10^9^/L)	11.51 (8.32‐15.05)	11.02 (8.66‐14.7)	11.87 (8.16‐15.46)	.751
Platelet (10^9^/L)	115 (76‐173.25)	91 (62‐138)	152 (102‐197)	<.001
Lymphocyte (10^9^/L)	0.83 (0.55‐1.15)	0.74 (0.52‐1.09)	0.93 (0.57‐1.23)	.046
Hemoglobin (g/L)	90 (76‐111.25)	81 (72‐99)	101 (84‐1221)	<.001
Albumin (g/L)	31.52 ± 7.76	28.78 ± 7.32	34.26 ± 7.23	<.001
Glucose (mmol/L)	9.67 (7.25‐13.02)	10.8 (8.42‐14.39)	8.31 (6.62‐11.6)	<.001
Potassium (mmol/L)	3.73 (3.31‐4.18)	3.68 (3.29‐4.2)	3.78 (3.38‐4.15)	.361
Phosphate (mmol/L)	0.84 (0.58‐1.16)	0.78 (0.46‐1.13)	0.91 (0.66‐1.17)	.007
Chlorine (mmol/L)	111.2 (105.7‐119.2)	116.9 (109.6‐126.3)	107.4 (103.4‐112.3)	<.001
Serum urea (mmol/L)	6.38 (5.02‐8.63)	7.11 (5.31‐9.8)	5.9 (4.57‐7.65)	<.001
Serum creatinine (umol/L)	75 (56‐99)	81 (60‐114)	68 (55‐87)	.001
RDW (%)	14.25 (13.3‐15.3)	15.3 (14.7‐16.2)	13.3 (12.9‐13.7)	<.001
Operations				
Decompressive craniectomy	96 (30.19%)	60 (37.74%)	36 (22.64%)	.003
Internal decompression	36 (11.32%)	21 (13.21%)	15 (9.43%)	.287
Hematoma evacuation	111 (34.91%)	67 (42.14%)	44 (27.67%)	.007
AKI	65 (20.44%)	50 (31.45%)	15 (9.43%)	<.001
In‐hospital mortality	158 (49.69%)	111 (69.81%)	47 (29.56%)	<.001
90‐d GOS	2 (1‐3)	1 (1‐2)	3 (1‐4)	<.001
Length of ICU stay (day)	2 (1‐18.25)	2 (1‐17)	3 (0‐19)	.569
Length of hospital stay (day)	12 (4.75‐29)	8 (3‐27)	18 (8‐30)	<.001

Note

All values are expressed as n (%) or median (first to third quartile).

Abbreviations: AKI, acute kidney injury; GCS, Glasgow Coma Scale; GOS, Glasgow Outcome Scale; MAP, mean arterial pressure; RDW, red blood cell distribution width; WBC, white blood cell.

### 3.2 Correlations between RDW and other variables

Performing Spearman's method, age, shock, GCS, platelet, albumin, glucose, serum urea, and creatinine were found to be weakly correlated with RDW (*P* < .001) (Table 2). There were moderate correlations between RDW and platelet, hemoglobin, albumin, chlorine, and 90‐day GOS (*P* < .001).

**Table 2:** Correlations between RDW and other clinical and laboratory variables

Variables	*r*	*P* value
Age	.201	<.001
Male	−.127	.024
MAP	−.009	.871
Shock	.262	<.001
GCS	−.231	<.001
WBC	.010	.854
Neutrophil	−.038	.496
Platelet	−.375	<.001
Lymphocyte	−.115	.040
Hemoglobin	−.408	<.001
Albumin	−.372	<.001
Glucose	.280	<.001
Potassium	−.045	.420
Phosphate	−.094	.093
Chlorine	. 416	<.001
Serum urea	.245	<.001
Serum creatinine	.202	<.001
90‐d GOS	−.445	<.001
Length of ICU stay	.068	.225
Length of hospital stay	−.179	.001

Abbreviations: GCS, Glasgow Coma Scale; GOS Glasgow Outcome Scale; MAP, mean arterial pressure; WBC, white blood cell.

### 3.3 Factors associated with AKI and mortality following TBI

In univariate logistic regression analysis, many variables were found associated with AKI and mortality including shock, GCS, chlorine, and RDW. (Table 3). However, the result of multivariate analysis showed that only lymphocyte (*P* = .012), chlorine (*P* = .028), serum creatinine (*P* < .001), and RDW (*P* = .034) were significant risk factors of AKI following TBI. Furthermore, GCS (*P* < .001), glucose (*P* = .007), chlorine (*P* = .004), RDW (*P* = .001), hematoma evacuation (*P* = .016), and AKI (*P* = .019) were significantly associated with mortality after adjustments (Table 4).

**Table 3:** Univariate and multivariate logistic regression analysis of factors associated with AKI following TBI

	Univariate analysis		Multivariate analysis	
	OR (95% Cl)	*P* value	OR (95% Cl)	*P* value
Age	1.015 (1.000‐1.030)	.052	1.019 (0.989‐1.050)	.219
Male	1.437 (0.722‐2.862)	.302	1.569 (0.438‐5.620)	.489
MAP	0.987 (0.973‐1.001)	.075	0.998 (0.984‐1.012)	.802
Shock	4.343 (2.462‐7.662)	<.001	2.255 (0.765‐6.644)	.140
GCS	0.753 (0.660‐0.858)	<.001	0.794 (0.610‐1.033)	.086
WBC	1.003 (0.963‐1.045)	.875	1.095 (0.991‐1.210)	.073
Neutrophil	0.994 (0.964‐1.024)	.670	0.926 (0.808‐1.062)	.273
Platelet	0.995 (0.991‐0.999)	.011	1.005 (0.997‐1.014)	.219
Lymphocyte	0.737 (0.462‐1.176)	.200	0.245 (0.082‐0.730)	.012
Hemoglobin	0.982 (0.970‐0.993)	.001	0.999 (0.978‐1.020)	.902
Albumin	0.934 (0.900‐0.969)	.001	1.021 (0.944‐1.103)	.605
Glucose	1.085 (1.029‐1.144)	.003	1.015 (0.933‐1.103)	.734
Potassium	1.261 (1.060‐1.502)	.009	1.346 (0.911‐1.988)	.136
Phosphate	1.092 (0.969‐1.232)	.150	0.933 (0.677‐1.287)	.673
Chlorine	1.058 (1.035‐1.082)	<.001	1.048 (1.005‐1.092)	.028
Serum urea	1.389 (1.254‐1.538)	<.001	1.147 (0.976‐1.348)	.095
Serum creatinine	1.049 (1.036‐1.063)	<.001	1.044 (1.026‐1.063)	<.001
RDW (%)	1.405 (1.215‐1.624)	<.001	1.398 (1.026‐1.905)	.034
Decompressive craniectomy	1.006 (0.572‐1.770)	.983	0.332 (0.081‐1.367)	.127
Internal decompression	0.916 (0.584‐1.437)	.702	1.625 (0.803‐3.285)	.177
Hematoma evacuation	1.050 (0.871‐1.265)	.612	1.161 (0.738‐1.826)	.518

Abbreviations: CI, confidence interval; GCS, Glasgow Coma Scale; MAP, mean arterial pressure; OR, odds ratio; RDW, red blood cell distribution width; WBC, white blood cell.

**Table 4:** Univariate and multivariate logistic regression analysis of factors associated with in‐hospital mortality after TBI

	Univariate analysis		Multivariate analysis	
	OR (95% Cl)	*P* value	OR (95% Cl)	*P* value
Age	1.004 (0.992‐1.016)	.497	0.999 (0.981‐1.016)	.881
Male	0.823 (0.488‐1.390)	.467	1.205 (0.531‐2.731)	.656
MAP	0.997 (0.989‐1.006)	.492	1.001 (0.990‐1.012)	.845
Shock	4.638 (2.732‐7.872)	<.001	0.806 (0.362‐1.797)	.599
GCS	0.628 (0.555‐0.710)	<.001	0.697 (0.596‐0.816)	<.001
WBC	1.025 (0.991‐1.060)	.155	1.014 (0.943‐1.091)	.702
Neutrophil	0.992 (0.971‐1.013)	.455	0.978 (0.896‐1.067)	.613
Platelet	0.992 (0.989‐0.995)	<.001	1.002 (0.997‐1.006)	.453
Lymphocyte	0.678 (0.477‐0.964)	.030	0.815 (0.478‐1.389)	.452
Hemoglobin	0.977 (0.967‐0.986)	<.001	1.001 (0.985‐1.017)	.914
Albumin	0.898 (0.868‐0.930)	<.001	0.960 (0.911‐1.012)	.132
Glucose	1.242 (1.161‐1.328)	<.001	1.132 (1.035‐1.239)	.007
Potassium	1.002 (0.863‐1.162)	.983	1.000 (0.735‐1.360)	1.000
Phosphate	0.999 (0.891‐1.120)	.983	0.987 (0.775‐1.256)	.913
Chlorine	1.113 (1.081‐1.146)	<.001	1.054 (1.017‐1.091)	.004
Serum urea	1.068 (1.013‐1.124)	.014	0.959 (0.883‐1.041)	.318
Serum creatinine	1.008 (1.004‐1.013)	.001	0.999 (0.992‐1.007)	.881
RDW (%)	1.770 (1.470‐2.132)	<.001	1.478 (1.165‐1.875)	.001
Decompressive craniectomy	1.682 (1.049‐2.698)	.031	1.756 (0.698‐4.418)	.232
Internal decompression	0.991 (0.698‐1.407)	.961	1.123 (0.633‐1.994)	.691
Hematoma evacuation	1.064 (0.912‐1.240)	.430	0.661 (0.472‐0.927)	.016
AKI	8.235 (4.015‐16.891)	<.001	3.989 (1.259‐12.635)	.019

Abbreviations: AKI, acute kidney injury; CI, confidence interval; GCS, Glasgow Coma Scale; MAP, mean arterial pressure; OR, odds ratio; RDW, red blood cell distribution width; WBC, white blood cell.

### 3.4 Predictive values of RDW in AKI and mortality

**Figure 2:**
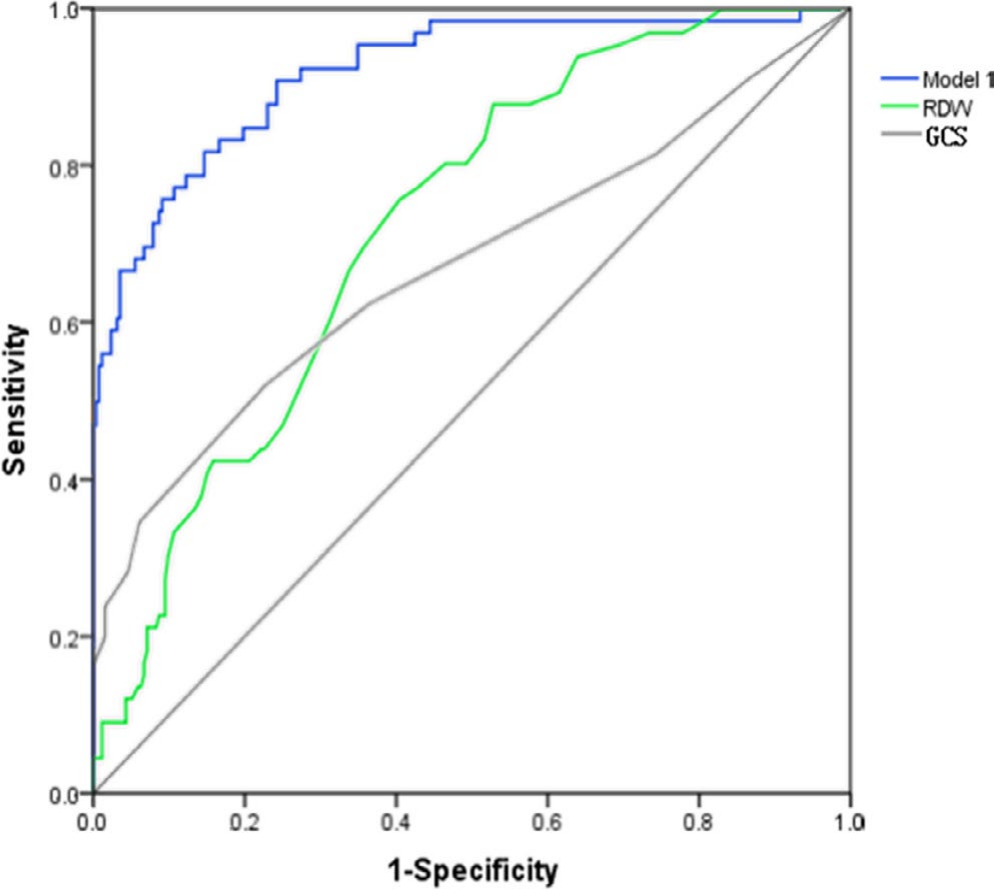
Receiver operating characteristic (ROC) curve analysis comparing the RDW, GCS, and model 1 for predicting AKI following TBI

**Figure 3:**
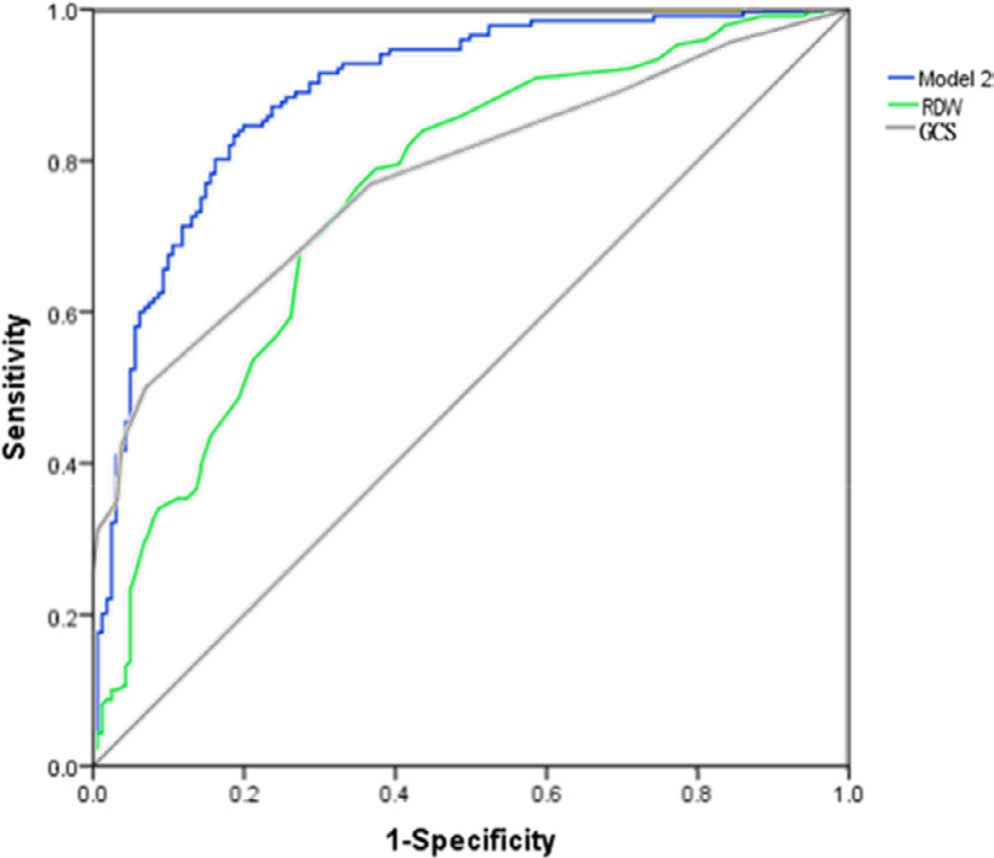
Receiver operating characteristic (ROC) curve analysis comparing the RDW, GCS, and model 2 for predicting in‐hospital outcome of patients with TBI

By drawing ROC curve, we calculated that the AUC of GCS to predict AKI was 0.672 (Figure 2). The sensitivity and specificity of GCS were 0.52 and 0.773, respectively (Table 5). The AUC of RDW to predict AKI was 0.724. The sensitivity, specificity, and cutoff value of RDW were 0.758, 0.595, and 14.35, respectively. The AUC of RDW was higher than GCS without statistical significance (*Z* = 1.1490, *P* > .05). Then, we used lymphocyte, chlorine, serum creatinine, and RDW which were statistically significant in multivariate analysis, to construct the model 1 to predict AKI. The AUC of model 1 was 0.919 with sensitivity of 0.818 and specificity of 0.853. In addition, the AUC of GCS to predict mortality was 0.778 with sensitivity of 0.500 and specificity of 0.930 (Table 6), while the AUC of RDW was 0.754 with cutoff value of 14, sensitivity of 0.791 and specificity of 0.625. RDW had comparable prognostic value with GCS (*Z* = 0.6403, *P* > .05). Significant factors which were obtained from multivariate analysis including GCS, glucose, chlorine, RDW, hematoma evacuation, and AKI were used to build the model 2 to predict mortality (Figure 3). The AUC of model 2 was 0.891 with sensitivity of 0.848 and specificity of 0.8.

**Table 5:** Comparisons of single RDW and models to predict AKI following TBI

	Sensitivity	Specificity	AUC (95% Cl)
GCS	0.520	0.773	0.672 (0.609‐0.735)
RDW (%)	0.758	0.595	0.724 (0.662‐0.786)
Model 1	0.818	0.853	0.919(0.880‐0.959)

Note

Model 1 consisted of lymphocyte, chlorine, serum creatinine, and RDW.

Abbreviations: AUC, area under the ROC curve; GCS, Glasgow Coma Scale; RDW, red blood cell distribution width.

**Table 6:** Comparisons of single RDW and models to predict in‐hospital mortality of patients with TBI

	Sensitivity	Specificity	AUC (95% Cl)
GCS	0.500	0.930	0.778 (0.727‐0.829)
RDW (%)	0.791	0.625	0.754 (0.701‐0.807)
Model 2	0.848	0.800	0.891 (0.856‐0.927)

Note

Model 2 consisted of GCS, glucose, chlorine, RDW, hematoma evacuation, and AKI.

Abbreviations: AUC, area under the ROC curve; GCS, Glasgow Coma Scale; RDW, red blood cell distribution width.

### 3.5 Association of RDW and survival time

**Figure 4:**
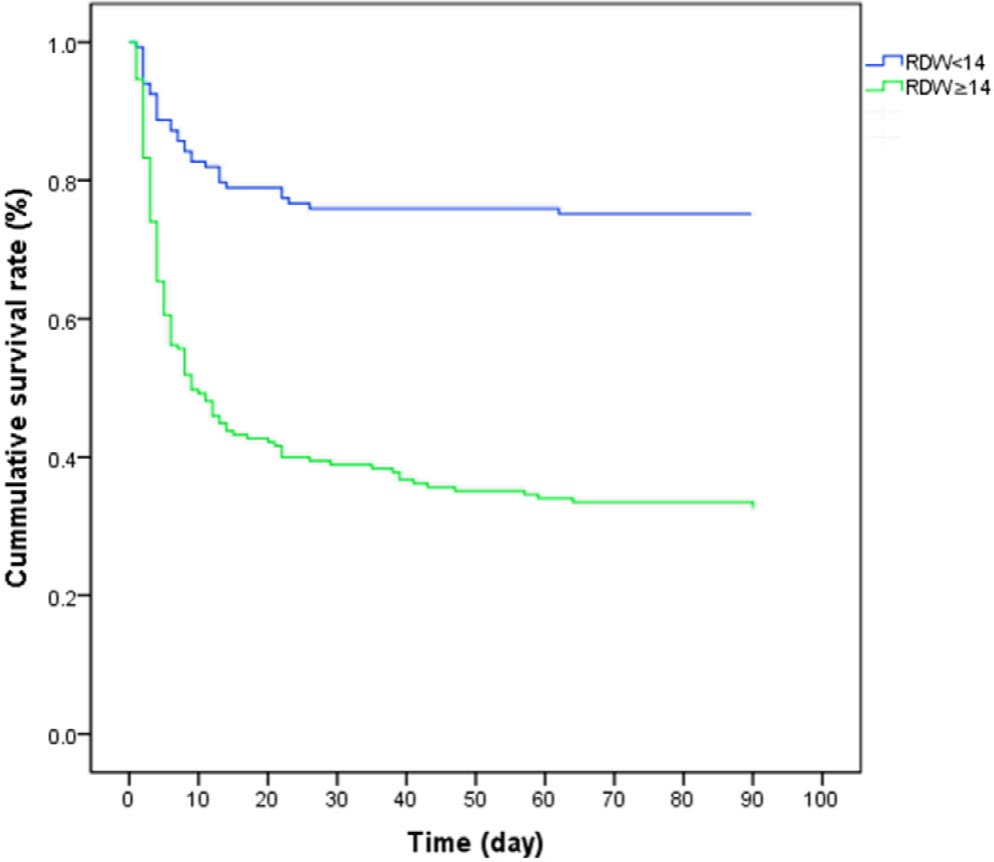
Kalpan‐Meir survival curves of patients with RDW ≥ 14 and RDW < 14. The mean survival of RDW ≥ 14 is 58 d and that of RDW < 14 is 70 d

We divided patients into two groups including whose RDW ≥ 14% and whose RDW < 14% according to the best cutoff value. Kaplan‐Meier survival curves showed that the survival time of high RDW group was significantly shorter than low RDW group (*P* < .001) (Figure 4).

## 4 DISCUSSION

Acute kidney injury following TBI has been paid much attention for the harmful effects it brings to the outcome. Several studies have investigated the incidence of AKI following TBI, ranged from 3.5% to 40%.^5^, ^6^, ^7^, ^19^, ^20^ The incidence of AKI was 20.44% in our study. This difference may be caused by the different diagnostic criteria of AKI over the years and respective medical level in different hospitals. It is now generally believed that non‐neurologic complications including AKI following TBI may be due to the massive catecholamine release, neuroinflammation, and side effects of therapies aimed at neuroprotection.^21^, ^22^

There were some studies exploring the association between elevated RDW and the development of AKI in several patient groups.^17^, ^18^, ^23^ One study showed that baseline RDW could predict the occurrence of AKI in patients treated in coronary care unit with a AUC value of 0.630. And the best cutoff value of RDW was 13.9.^18^ Another study found that RDW was independently correlated with contrast‐induced AKI and was valuable in predicting AKI with a AUC value of 0.663 in patients undergoing a primary percutaneous coronary intervention.^23^ Our study showed that RDW was a predictor of AKI following TBI with AUC of 0.724. The underlying mechanism between RDW and AKI has not been thoroughly clarified. There are several explanations for the association of RDW and AKI following TBI. Firstly, inflammation reflected by RDW might play an important role in the development of AKI. Previous study has confirmed that elevation of inflammatory cytokines including TNF‐α, IL‐1β, and IL‐6 could inhibit erythropoietin (EPO)‐induced erythrocyte (RBC) maturation.^24^, ^25^, ^26^ Meanwhile, the RDW would rise above normal level, reflecting an increase in the proportion of juvenile RBCs.^27^ TBI can trigger a massive release of inflammatory mediators including TNF‐α, IL‐1β, and IL‐6, CRP, which can accelerate neuroinflammation and systemic organ damage.^28^ In addition, the development of AKI is associated with intrarenal and systemic inflammation.^29^ One study has validated the predictive value of serum CRP, a common inflammatory marker, in AKI following aneurysmal subarachnoid hemorrhage.^30^ Therefore, the RDW might be associated with AKI following TBI through the mediation of systemic inflammation. Secondly, oxidative stress may contribute to anisocytosis through inhibiting erythropoiesis and impairing red blood cell membrane deformability which in turn shorten the circulation half‐life of red blood cell and result in an increase in RDW value.^31^, ^32^ Furthermore, the triangle among reactive oxygen species (ROS) and nitric oxide (NO), and oxygen was involved in the oxidative stress system and play an important role in the pathogenesis of AKI.^33^, ^34^ Thus, RDW may be valuable in evaluating the oxidative stress status in kidney. Thirdly, it has been illustrated that the sympathetic system and the renin‐angiotensin system (RAAS) may promote the release of erythropoietin. Consequently, erythropoiesis is accelerated and the heterogeneity of red blood cell increased.^35^ The catecholamine surge after TBI may contribute to non‐neurologic complications including AKI.^21^ We make a reasonable conjecture that the increase of RDW after TBI may reflect the activity of sympathetic nervous system and level of plasma catecholamine including norepinephrine, epinephrine, and dopamine. Finally, it has been reported that elevated RDW is associated with malnutrition.^36^ And hypoalbuminemia, a marker of malnutrition, is an independent risk factor of AKI in various clinical settings.^37^, ^38^, ^39^ Therefore, malnutrition is another bridge to understand the relationship between elevated RDW and AKI.

Previous studies had explored the prognostic value of RDW in TBI patients with contradictory results. One study demonstrated that RDW was a poor prognostic marker of mortality with a AUC value of 0.66 in TBI patients.^14^ However, another study confirmed that RDW was a strong indicator of mortality with a AUC value of 0.805 in a small group of TBI patients.^15^ And neither of these studies analyzed the association between RDW and mortality in multivariate logistic regression. Our study confirmed that RDW was indeed a predictor of mortality in TBI patients with a AUC value of 0.754. Mechanisms of mediating AKI and RDW mentioned above also play an important role in the development of secondary brain injury and clinical outcome after TBI, including local neuroinflammation and systemic inflammation, activation of oxidative stress system and sympathetic system, and malnutrition.^40^, ^41^, ^42^, ^43^ And markers of these mechanisms have been confirmed to be valuable in predicting outcome of patients with TBI, including catecholamine, and albumin.^44^, ^45^, ^46^ The association between increased RDW and unfavorable outcome may be mediated through these mechanisms. In addition, increased RDW means that an increasing number of red blood cells with partially saturated hemoglobin. One study inferred that elevated RDW reflected decreased oxygen transport capacity.^47^ It is well known that adequate oxygen delivery is essential for cerebral metabolism and recovery of patients with TBI. Excessively high value of RDW can cause the insufficiency of cerebral oxygen and then worsen the outcome.

In addition to RDW, we found that lymphocyte, chlorine, and serum creatinine were also risk factors of AKI in model 1. Recent evidence demonstrates that both innate and adaptive immune cells play an important role in initiating and promoting damage to renal tubular in the development of AKI.^48^ In addition, there are several studies confirming that high neutrophil lymphocyte ratio is independently associated with the development of AKI in some clinical settings.^49^, ^50^, ^51^ The OR value of lymphocyte in multivariate logistic regression analysis is 0.245, which indicates that increased lymphocyte is a protective factor in the development of AKI. However, this result is worth further exploring in future study because of diverse role of different immune cells in AKI including monocytes, neutrophils, T lymphocytes, and B lymphocytes.^52^ Hyperchloremia has been validated valuable in predicting AKI in patients undergoing craniotomy for brain tumor resection and those diagnosed with subarachnoid hemorrhage.^53^, ^54^, ^55^, ^56^ This fact emphasized that physicians should pay attention to AKI when regularly using 0.9% normal saline to reduce intracranial pressure.

### 4.1 Study limitations

There are several limitations in our study. First, the retrospective and single‐center study design leads to the fact that selection bias was unavoidable. Second, hematopoietic raw material including iron, vitamin B12, and folate was not measured and history of hemolysis or blood transfusion was not recorded. These factors might influence the value of RDW. Third, as mentioned above, inflammation and activation of sympathetic system and RAAS might contribute to the development of AKI and unfavorable outcome after TBI. However, we did not measure well‐known markers of these systems including C‐reactive protein, norepinephrine, and angiotensin II. Finally, stages of AKI were not recorded by us so that we could not analyze the exact association between RDW and the severity of AKI.

## 5 CONCLUSION

This study suggests that RDW is valuable in predicting AKI following TBI and is an efficient and economical predictor of in‐hospital outcome in patients with TBI.

## CONFLICT OF INTEREST

The authors declared that they have no conflicts of interest to this work.

## AUTHOR CONTRIBUTIONS

Ruoran Wang and Min He designed this clinical study. Xiaofeng Ou and Xiaoqi Xie collected all the data. Ruoran Wang analyzed the data and wrote the drafts of the article. Yan Kang edited and reviewed drafts of the article.
